# Assessment of Hepcidin-25 and Iron Status Profiles in Pregnant Women With Thalassemia Minor

**DOI:** 10.1155/jp/6150362

**Published:** 2025-11-07

**Authors:** Thunthida Jiampochaman, Theera Tongsong, Somdet Srichairatanakool, Pimpisid Koonyosying, Narisara Paradee, Onsaya Kerdto, Suchaya Luewan

**Affiliations:** ^1^Department of Obstetrics and Gynecology, Faculty of Medicine, Chiang Mai University, Chiang Mai, Thailand; ^2^Department of Biochemistry, Faculty of Medicine, Chiang Mai University, Chiang Mai, Thailand

**Keywords:** anemia, hemoglobin, hepcidin, iron, pregnancy, thalassemia minor, transferrin receptor

## Abstract

**Objective:**

The objective of the study is to compare hepcidin-25 levels between normal pregnant women and those with thalassemia minor.

**Methods:**

This prospective cohort study involved pregnant women with either normal pregnancies or thalassemia minor. Hepcidin-25 levels and iron study panels were measured at three time points: in the first trimester before the start of iron supplementation (gestational age [GA] < 14 weeks), in the third trimester (GA 28–32 weeks), and after GA 36 weeks.

**Results:**

The study included 125 pregnant women, comprising 93 with normal pregnancies and 32 with thalassemia minor. The hepcidin levels in the thalassemia minor group at GA 28–32 weeks and after GA 36 weeks were significantly lower than those in the normal pregnancy group (*p* values < 0.01 and 0.01, respectively). The study group exhibited mild anemia and lower Hb levels throughout pregnancy compared with the control group.

**Conclusion:**

Hepcidin-25 levels are significantly lower in pregnant women with thalassemia minor, but other iron profiles in these women are comparable to those in normal pregnancies, with no evidence of iron overload. Pregnancy with thalassemia minor is associated with mild anemia that cannot be fully corrected by iron supplementation. However, iron supplementation does not lead to iron overload and should be prescribed as part of standard antenatal care.

**Summary**

Hepcidin-25 (Hcd) levels are significantly lower in pregnant women with thalassemia minor, but other iron profiles in these women are comparable to those in normal pregnancies.

## 1. Introduction

Thalassemia, an autosomal recessive hereditary disorder, represents a significant global health concern, particularly in regions with high prevalence, such as Southeast Asia. In Thailand, the estimated prevalence of thalassemia is approximately 20%–30% for alpha-thalassemia and 3.0%–9.0% for beta-thalassemia [1, 2]. During pregnancy, the maternal demand for iron increases substantially to support the enhanced production of red blood cells, which is critical for placental and fetal development. Therefore, major health organizations, including the World Health Organization (WHO) and the American College of Obstetricians and Gynecologists (ACOG), recommend iron supplementation for all pregnant women. However, caution must be exercised when prescribing iron supplements to women with thalassemia, as they are predisposed to iron overload, which can lead to serious long-term adverse outcomes. Women with thalassemia minor, including alpha-thalassemia trait, beta-thalassemia trait, and hemoglobin (Hb) E trait [2], can also exhibit subclinical iron elevations, in spite of no blood transfusion. The mechanisms underlying iron loading in these individuals are often linked to increased intestinal absorption due to ineffective erythropoiesis and hepcidin suppression, commonly observed in nontransfusion-dependent thalassemia (NTDT) [3]. Whether iron supplementation is necessary in this population remains a subject that requires further investigation.

Hepcidin, a 25-amino acid peptide produced by hepatocytes, has emerged as the key regulator of iron uptake and release in tissues, ensuring a steady supply of iron to the erythron and other organs while preventing excessive levels that could be detrimental [4–7]. Hepcidin regulation is influenced by iron availability, the demand for erythropoiesis, and the inflammatory state [4–6]. Elevated iron levels or inflammation stimulate hepcidin synthesis in the liver, leading to reduced ferroportin expression, decreased intestinal iron absorption, and a consequent drop in circulating iron levels. Conversely, reduced systemic iron availability inhibits hepcidin production, promoting increased intestinal iron absorption and higher serum iron (SI) levels. During pregnancy, hepcidin levels progressively decline with advancing gestation to meet the increasing iron requirements of both the mother and the fetus [8].

In patients with thalassemia, ineffective erythropoiesis results in increased iron absorption and accumulation in tissues, often exceeding the demand for erythropoiesis. The mechanisms underlying iron overload are probably associated with suppression of hepcidin [9]. Chen et al. [10] investigated hepcidin levels in pregnant women who were beta-thalassemia carriers compared to those with normal pregnancies. Their findings indicated that nonanemic carriers exhibited hepcidin levels similar to those seen in normal pregnancies, while carriers with iron deficiency had hepcidin levels comparable to iron-deficient women without thalassemia. However, this study did not stratify participants by gestational age, as it included all trimesters without subgroup analysis.

To date, research on hepcidin levels in pregnant women with thalassemia remains limited. Therefore, we conducted this study to investigate iron profiles in normal pregnancies and those complicated by thalassemia minor. The primary objective is to compare hepcidin levels between normal pregnant women and those with thalassemia minor and to examine hepcidin changes throughout pregnancy in both groups. The secondary objectives include comparing red blood cell indices and iron profiles, such as serum ferritin, SI, transferrin saturation, and transferrin receptors, between normal pregnancies and those complicated by thalassemia minor.

## 2. Patients and Methods

This prospective cohort study was conducted on normal pregnant women and women with thalassemia minor at Maharaj Nakorn Chiang Mai, a university hospital and tertiary medical center. The study was ethically approved by the Institutional Review Board (Committee 4, Faculty of Medicine, Chiang Mai University, Thailand), Research ID OBG-2565-9193, date of approval: 4 November 2022. The study population comprised pregnant women attending our antenatal care clinic between September 2022 and August 2024. Eligible patients were invited to participate, and all participants provided written informed consent.

Inclusion criteria were as follows: (1) age 20 years or older; (2) singleton pregnancy; and (3) gestational age less than 14 weeks, confirmed by a reliable last menstrual period and first-trimester ultrasound of fetal crown-rump length. Participants were divided into two groups: (1) Study group: pregnant women with thalassemia minor, including alpha^0^-thalassemia trait, beta-thalassemia trait, and Hb E trait. Diagnosis was confirmed by Hb typing using high-performance liquid chromatography (HPLC) and polymerase chain reaction (PCR) for alpha^0^-thalassemia Southeast Asian type (SEA type). (2) Control group: normal pregnant women in the first trimester and without any underlying medical conditions.

Exclusion criteria included women diagnosed with anemia due to other causes, such as hematologic disorders (e.g., G6PD deficiency and thalassemia major), immunological compromise, autoimmune disease, peptic ulcer, severe infection, liver impairment, or chronic diseases. Withdrawal criteria included patients who developed other illnesses preventing them from continuing in the study, those unable to follow the protocol in a manner that might significantly affect data reliability, and those who chose to withdraw from the study.

Participants were thoroughly briefed on the study protocol, and comprehensive demographic data, including medical and obstetric histories, were collected. Blood samples were obtained from each participant at three distinct time points: (1) At recruitment, with a gestational age of no more than 14 weeks, an additional 5 mL of blood was drawn for assessment of iron status, including hepcidin, transferrin receptor, soluble ferritin (sFt), SI, and total iron-binding capacity (TIBC), alongside routine antenatal screening including iron supplement (daily oral ferrous fumarate 200 mg, equivalent to approximately 65 mg of elemental iron); (2) during the early third trimester (28–32 weeks' gestation), an additional 5-mL blood sample was collected in conjunction with routine antenatal laboratory tests; and (3) after 36 weeks' gestation or at the onset of labor. All participants received standard antenatal, intrapartum, and postpartum care in accordance with established clinical guidelines.

Laboratories for iron studies are summarized as follows:

1. *Serum hepcidin determination:* The quantification of serum Hcd was conducted using a sandwich enzyme-linked immunosorbent assay (sandwich ELISA). Quality control assessments of the assay indicated a sensitivity of < 0.12 ng/L, with intra-assay precision (coefficient of variation [CV] < 10%) and interassay precision (CV < 12%).
2. *Soluble transferrin receptor 1 (sTfR-1) determination:* The quantification of serum sTfR-1, CD71, was conducted using a sandwich ELISA, adhering to the protocol provided by ABBEXA Company Limited (Bar Hill, Cambridge, United Kingdom). Quality control assessments demonstrated a sensitivity of 0.94 ng/L, with intra-assay precision (CV < 10%) and interassay precision (CV < 10%).
3. *Determination of sFt Concentration:* Serum sFt was quantified using an electrochemiluminescence immunoassay (ECLIA) [11] based on Elecsys technology. The assay was performed on a Cobas Roche Automated ChemAnalyzer, in accordance with the manufacturer's protocol, as provided by Roche Diagnostics International AG, Rotkreuz, Switzerland.
4. *Determination of SI and unsaturated iron-binding capacity (UIBC):* The quantifications of SI and UIBC were performed using the ferrozine colorimetry [12] on a Cobas Roche Automated ChemAnalyzer, in accordance with the manufacturer's protocol provided by Roche Diagnostics International AG, Rotkreuz, Switzerland. TIBC was calculated using the equation:

(1) $$\begin{array}{c} \text{TIBC}=\text{SI}+\text{UIBC} \end{array}$$

Accordingly, transferrin saturation (%TS) was subsequently calculated using the equation:

(2) $$\begin{array}{c} \%\text{TS}=\left(\text{SI}/\text{TIBC}\right)\times 100 \end{array}$$

The primary outcomes of the study involved comparing the concentrations of hepcidin, sFt, SI, TIBC, and sTfR-1 between normal pregnant women and those with thalassemia minor.

### 2.1. Statistical Analysis

Statistical analyses were conducted using the Statistical Package for the Social Sciences (SPSS) software, Version 26.0 (IBM Corp., Released 2019). Comparisons of baseline characteristics, hematologic parameters, and iron profiles between the two groups were performed using either Student's *t*-test or the Mann–Whitney *U* test for continuous variables, depending on the normality of distribution, and the chi-square test for categorical variables. Comparisons of parameters across the three trimesters were analyzed using the Kruskal–Wallis test. Trends in hematologic and iron parameters within each group over time were assessed using repeated measures ANOVA. A *p* value of < 0.05 was considered statistical significance.

## 3. Results

During the study period, a total of 125 cases were recruited and met the inclusion criteria: 93 cases in the control group (normal) and 32 cases in the study group (thalassemia minor), which included three subgroups: alpha^0^-thalassemia trait (11 cases), beta-thalassemia trait (11 cases), and Hb E trait (10 cases). Baseline demographic data, including maternal age, prepregnancy weight, and parity, did not differ significantly between the two groups, as shown in Table 1.

However, several baseline hematologic parameters from the first visit were significantly different between the groups, as detailed in Table 2. The study group exhibited mild anemia with microcytosis compared to the control group (Hb level: 11.2 ± 0.8 vs. 12.7 ± 0.8 g/dL, respectively; *p* < 0.001). Despite this, iron profiles, including SI, TIBC, TS, sFt, and sTfR-1 levels, were comparable and within normal limits in both groups. Baseline hepcidin levels at the first visit were lower in the study group but did not reach statistical significance (21.67 vs. 25.03 ng/mL, respectively; *p* = 0.07).

Comparisons of hematologic parameters from the second and third lab tests also revealed significant differences between the groups, consistent with the first lab tests. However, iron profiles remained comparable in most parameters. Notably, the study group continued to have lower Hb levels in the second test despite receiving iron supplements (10.7 ± 1.0 vs. 11.9 ± 0.8 g/dL, respectively; *p* < 0.001). Hepcidin levels in the study group were significantly lower in the second lab test (16.29 vs. 23.90 ng/mL, respectively; *p* < 0.001) and showed an even greater difference in the third lab test (15.93 vs. 27.07 ng/mL, respectively; *p* < 0.001). Additionally, transferrin receptor levels, which tended to be higher (though not significantly) in the first and second tests, became slightly, but significantly, higher in the study group in the third lab test.

Subgroup analysis indicated that Hb E trait was associated with less severe anemia in terms of Hb levels. However, levels in all three subgroups were significantly lower compared to normal pregnancies, although other iron profiles were similar between the subgroups and the normal group, as presented in Table 3.

**Figure 1:**
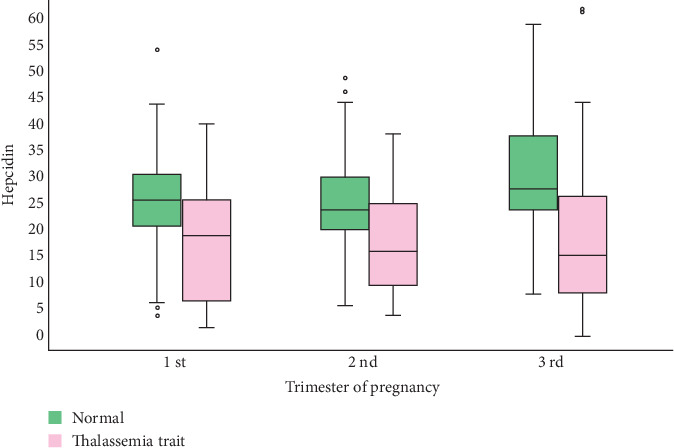
Boxplots of hepcidin-25 levels at the three points of pregnancy of the two groups (groups and time points are significant, independent factors of hepcidin levels, *p* < 0.01).

As a longitudinal study, hepcidin levels in normal pregnancies showed a trend of decreasing during the early third trimester and rising at term or just before labor. In contrast, hepcidin levels in the study group tended to decrease throughout gestation, as presented in Table 4. Both gestational age and thalassemia status were significant independent factors influencing hepcidin levels in the multivariable analysis (repeated measures ANOVA). The magnitude of hepcidin change between the two groups was also significant, as illustrated in Figure 1.

Obstetric outcomes were comparable between the two groups. Rates of small for gestational age, preterm birth, low birth weight, and low Apgar scores at 1 and 5 min did not differ significantly. Maternal weight gain and birth weight were also comparable. However, the prevalence of gestational diabetes mellitus (GDM) was significantly higher in the study group (10.75% vs. 34.38% in the control and study groups, respectively; *p* < 0.001).

## 4. Discussion

Insights gained from this study are as follows: (1) Although thalassemia minor is associated with mild anemia, women with this condition show lower Hb levels than those with normal pregnancies throughout gestation. Among these, the Hb E subgroup is the least severe. (2) Iron supplementation does not correct anemia associated with thalassemia minor; however, standard iron supplementation does not lead to iron overload. (3) Anemia associated with thalassemia minor is not related to iron deficiency. (4) In early pregnancy, hepcidin levels in women with thalassemia minor are slightly lower but not significantly different and the levels are significantly lower throughout the remainder of the pregnancy. However, the levels in normal pregnancies had a trend to increase in late gestation. The gap in hepcidin levels between the two groups widens as gestational age increases. (5) Hematologic parameters show significant differences throughout pregnancy despite iron supplementation. (6) All iron profiles, except for hepcidin, are comparable between the two groups throughout pregnancy. (7) The three types of thalassemia minor exhibit similar iron profiles and transferrin receptor levels, all of which show lower hepcidin levels than in normal pregnancies throughout pregnancy.

Although iron supplementation is a standard practice in pregnancy care, iron overload is a concern in women with thalassemia due to the potential for increased intestinal absorption caused by ineffective erythropoiesis and suppressed hepcidin levels [3]. However, the findings of this study show that despite a further reduction in hepcidin levels, which theoretically increases the risk of iron overload, patients maintained normal iron profiles. There was no evidence of iron deficiency or overload, even with iron supplementation, though mild anemia persisted. Therefore, lower hepcidin levels in pregnant women with thalassemia minor may not be a sign of excessive iron accumulation. As a result, thalassemia minor should not be a cause for concern regarding iron overload, and standard antenatal care guidelines for iron supplementation should be followed. The exact mechanism behind the reduction in hepcidin remains unclear, but it does not seem to pose a risk of iron overload. However, it should be emphasized that all subgroups of thalassemia minor exhibited mild anemia throughout pregnancy, and iron supplementation did not improve Hb levels. Also, it is important to note that hematologic parameters in the study group were lower than those in normal pregnancies, both in early pregnancy (prior to supplementation) and in the early third trimester (after adequate iron supplementation), despite the absence of iron deficiency.

This study demonstrated that both pregnancy and thalassemia minor independently contribute to the suppression of hepcidin. Despite iron supplementation, women with thalassemia minor exhibited significantly lower hepcidin levels compared to those with normal pregnancies, with the difference becoming more pronounced in late gestation. The finding that hepcidin levels were significantly higher during early labor in this study aligns with the results reported by Hedengran et al. [13], which demonstrated a significant increase in hepcidin levels during delivery, likely functioning as a marker of inflammation. However, in women with thalassemia minor, hepcidin levels were much lower compared to those in normal pregnant women, while other markers of iron metabolism were comparable. This suggests ineffective erythropoiesis in thalassemia minor without being associated with iron overload or deficiency.

Persistently low Hb levels throughout pregnancy, despite iron supplementation, may explain the increased risk of adverse pregnancy outcomes in women with thalassemia minor, as reported in previous studies [14–18]. In addition, physiological changes during pregnancy may worsen the severity of anemia in pregnant women with thalassemia traits. However, pregnancy outcomes in this study were comparable to those in normal pregnancies. This is likely due to the small sample size, which may have been insufficient to detect the small effect size on adverse outcomes.

The transferrin receptor, which mediates cellular iron uptake through clathrin-dependent endocytosis of iron-loaded transferrin, plays a key role in iron homeostasis. Since the number of transferrin receptors on the cell surface is the rate-limiting step for iron entry into cells and is essential for preventing iron overload [19], it is noteworthy that the levels of transferrin receptors in pregnant women with thalassemia minor are not significantly different from those in normal pregnant women.

### 4.1. Clinical Implication

This study provides evidence that iron supplementation in women with thalassemia minor does not lead to iron overload, supporting the use of iron supplementation similar to that in normal pregnancies to prevent further anemia. However, the significantly lower hepcidin levels reflect ineffective erythropoiesis, which cannot be fully corrected by iron supplementation, and Hb concentrations in women with thalassemia minor remain lower. Due to their lower Hb levels and the inability to improve them with iron supplementation, even though the anemia is mild, these women should receive special care. They are at higher risk of adverse pregnancy outcomes and are more vulnerable to excessive blood loss, particularly postpartum hemorrhage, compared to women with normal pregnancies.

### 4.2. Research Implication

Pregnant women with thalassemia intermedia or major should also be studied for their iron profiles, as this group may have excessive iron. It is not yet clear whether iron supplementation during pregnancy is as necessary for them as it is for women with normal pregnancies. Additionally, subgroup analyses should be conducted to address the different profiles among the various thalassemia subtypes. It remains to be determined whether lower or higher hepcidin levels during pregnancy are pathogenic factors that adversely impact obstetric outcomes. Interestingly, we observed that the prevalence of GDM increased in the study groups. The reason for this observation remains unclear and further studies are required. However, it is possible that patients with thalassemia trait had some degree of subclinical iron overload, which may promote oxidative damage through the Fenton reaction, thereby increasing the incidence of gestational diabetes. This is consistent with the established understanding that iron-mediated oxidative stress is a central mechanism impairing glucose metabolism and insulin sensitivity.

### 4.3. Weaknesses

(1) Although the overall sample size appears adequate to address the primary objective, the sample size for subgroups is too small to determine whether the iron profiles among these subgroups differ significantly. Accordingly, the sample size may be inadequate for certain comparisons and the findings should be interpreted with caution. (2) Hcd levels are certainly affected by iron supplementation, which may obscure the direct effects of pregnancy on hepcidin levels. This limitation could not be avoided, as it would be unethical not to prescribe iron supplementation to pregnant women, given that it is a standard part of antenatal care. Additionally, potential confounding factors, such as variations in dietary intake and the presence of inflammation from any cause, were not comprehensively assessed.

### 4.4. Strengths

(1) The study focuses not only on comparing hepcidin levels but also on evaluating other hematological parameters and iron profiles in the two groups. (2) It examines not only the overall changes in various parameters but also the longitudinal trends at three different time points during pregnancy.

### 4.5. Conclusion

Hepcidin levels are significantly lower in pregnant women with thalassemia minor, indicating ineffective erythropoiesis. Despite the marked reduction in hepcidin levels, other iron profiles in these women are comparable to those in normal pregnancies, with no evidence of iron overload. Pregnancy with thalassemia minor is associated with mild anemia that cannot be fully addressed by iron supplementation. However, iron supplementation does not lead to iron overload and should be prescribed as part of standard antenatal care.

## Figures and Tables

**Table 1:** Demographic data and obstetric outcomes of the two groups.

	**Normal (** **n** = 93**)**	**Thalassemia trait (** **n** = 32**)**	**p** ** value**
Age (year): Mean ± SD	31.69 ± 4.16	30.16 ± 4.82	0.08
Subgroup: *n* (%)			
Normal	93 (100.00)	0 (0.00)	< 0.01
Alpha-thalassemia trait	0 (0.00)	11 (34.38)	< 0.01
Beta-thalassemia trait	0 (0.00)	11 (34.38)	< 0.01
HB E trait	0 (0.00)	12 (37.50)	< 0.01
Prepregnancy weight (kg): median (q1–q3)	55 (49–64)	55 (49.50–65)	0.63
Parity: *n* (%)			0.29
Nulliparity	48 (51.61)	20 (62.50)	
Multiparity	45 (48.39)	12 (37.50)	
Gestational age (GA) (weeks) 1st: Median (q1–q3)	11 (8–12)	12 (10.50–13)	0.01
Weight 1st: Median (q1–q3)	56.10 (51.20–64.70)	54.55 (50.15–68.25)	0.98
Weight (kg) 1st, different from prepregnancy: Mean ± SD	1.12 ± 1.93	0.73 ± 3.14	< 0.01
GA 2nd: Median (q1–q3)	29 (28–29)	29 (28–30)	0.47
Weight 2nd: Median (q1–q3)	64.60 (59.10–71.50)	63.05 (58.95–76.40)	0.86
Difference between 2nd and 1st weight (kg): Mean ± SD	7.34 ± 2.95	8.04 ± 3.316	0.27
Difference between 2nd and prepregnancy weight (kg): Mean ± SD	8.46 ± 3.61	8.77 ± 3.88	0.68
Weight 3rd: Median (q1–q3)	68 (62–75)	65.60 (59.70–78)	0.69
Difference between 3rd and prepregnancy weight (kg): Mean ± SD	12.21 ± 4.09	11.75 ± 4.46	0.64
Difference weight between 3rd and 2nd (kg): Median (q1–q3)	3.70 (2.60–5)	3.20 (2.20–4.60)	0.31
Newborn birthweight, percentile: *n* (%)			> 0.99
Normal	65 (91.55)	21 (91.30)	
Small-for-gestational age	6 (8.45)	2 (8.70)	
Large-for-gestational age	0 (0.00)	0 (0.00)	
Apgar score at 1 min: Median (q1–q3)	9 (8–9)	8 (8–9)	0.70
Apgar score at 5 min: Median (q1–q3)	10 (9–10)	9 (9–10)	0.86
Apgar score at 10 min: Median (q1–q3)	10 (10–10)	10 (10–10)	0.37
Gestational diabetes mellitus (GDM): *n* (%)			< 0.01
No GDM	81 (87.10)	20 (62.50)	< 0.01
GDM	10 (10.75)	11 (34.38)	< 0.01
Overt diabetes mellitus	2 (2.15)	1 (3.13)	0.76

**Table 2:** Hematologic studies and iron profiles of the two groups.

	**Normal (** **n** = 93**)**	**Thalassemia trait (** **n** = 32**)**	**p** ** value**
**Gestational age (GA) (weeks) 1st: Median (q1–q3)**	**11 (8–12)**	**12 (10.50–13)**	**0.01**
Hb (g/dL) 1st: Mean ± SD	12.71 ± 0.82	11.19 ± 0.86	< 0.01
Hct (%) 1st: Mean ± SD	38.77 ± 2.55	34.84 ± 2.77	< 0.01
MCV (fL) 1st: Mean ± SD	88.18 ± 5.05	67.78 ± 7.46	< 0.01
MCHC (g/dL) 1st: Mean ± SD	32.78 ± 0.95	32.13 ± 1.13	< 0.01
RBC (×10^6^ cells/*μ*L) 1st: Mean ± SD	4.61 ± 0.40	5.16 ± 0.63	< 0.01
Serum hepcidin-25 (ng/mL) 1st: Median (q1–q3)	25.03 (20.12–30.08)	21.67 (8.94–29.76)	0.07
SI (*μ*g/dL) 1st: Median (q1–q3)	91.84 (75.91–112.02)	89.30 (76.58–106.24)	0.45
TIBC (mg/dL) 1st: Mean ± SD	262.86 ± 46.11	262.64 ± 45.05	0.98
TSAT (%) 1st: Median (q1–q3)	33.68 (28.15–40.39)	34.38 (29.96–43.62)	0.81
sFt (ng/mL) 1st: Median (q1–q3)	119.30 (70.02–177.60)	126.60 (63.03–175.30)	0.80
sTfR-1 (ng/mL) 1st: Median (q1–q3)	5204.25 (4784.66–5682.16)	5498.06 (4958.91–5853.80)	0.14

**GA 2nd: Median (q1–q3)**	**29 (28–29)**	**29 (28–30)**	**0.47**
Hb (g/dL) 2nd: Mean ± SD	11.94 ± 0.76	10.74 ± 0.96	< 0.01
Hct (%) 2nd: Mean ± SD	36.10 ± 2.51	33.77 ± 2.75	< 0.01
MCV (fL) 2nd: Mean ± SD	89.97 ± 5.19	70.61 ± 7.64	< 0.01
MCHC (g/dL) 2nd: Mean ± SD	33.11 ± 0.99	31.74 ± 1.00	< 0.01
RBC (×10^6^ cells/*μ*L) 2nd: Mean ± SD	4.03 ± 0.40	4.79 ± 0.47	< 0.01
Serum hepcidin-25 (ng/mL) 2nd: Median (q1–q3)	23.90 (19.56–30.48)	16.29 (9.63–21.59)	< 0.01
SI (*μ*g/dL) 2nd: Median (q1–q3)	76.53 (58.64–90.33)	78.71 (61.69–95.06)	0.47
TIBC (mg/dL) 2nd: Median (q1–q3)	330.30 (297.66–373.18)	339.54 (301.78–389.79)	0.33
TSAT (%) 2nd: Median (q1–q3)	21.64 (17.2–28.84)	22.53 (18.88–28.03)	0.67
sFt (ng/mL) 2nd: Median (q1–q3)	56.50 (41.38–92.88)	56.34 (35.87–100.95)	0.80
sTfR-1 (ng/mL) 2nd: Mean ± SD	5372.04 ± 609.19	5524.13 ± 480.62	0.20

**GA 3rd: Median (q1–q3)**	**36–40**	**36–40**	**0.61**
Serum hepcidin-25 (ng/mL) 3rd: Median (q1–q3)	27.07 (22.16–36.23)	15.93 (10.97–29.69)	0.01
SI (*μ*g/dL) 3rd: Median (q1–q3)	82.59 (61.77–109.23)	102.35 (67.69–135.78)	0.07
TIBC (mg/dL) 3rd: Mean ± SD	332.25 ± 71.67	341.4 ± 49.24	0.57
TSAT (%) 3rd: Median (q1–q3)	25.50 (18.62–35.64)	26.42 (21.01–43.48)	0.34
sFt (ng/mL) 3rd: Median (q1–q3)	97.49 (70.22–126.50)	101.90 (57.46–166.50)	0.96
sTfR-1 (ng/mL) 3rd: Mean ± SD	5228.95 ± 608.79	5531.27 ± 457.99	0.03

Abbreviations: GA, gestational age; Hb, hemoglobin; Hct, hematocrit; MCH, mean corpuscular hemoglobin; MCHC, mean corpuscular hemoglobin concentration; MCV, mean corpuscular volume; RBC, red blood cell; sFT, soluble ferritin; SI, serum iron; sTfR-1, soluble transferrin receptor 1; TIBC, total iron-binding capacity; TSAT, transferrin saturation.

**Table 3:** Comparisons of iron profiles among normal pregnant women and the three subgroups of pregnant women with thalassemia trait.

	**Normal (** **n** = 93**)**	**Alpha-thal trait (** **n** = 9**)**	**Beta-thal trait (** **n** = 11**)**	**HB E trait (** **n** = 10**)**	**p** ** value**
Lab assessment at first visit					
Serum hepcidin-25 (ng/mL) lab 1st: Median (q1–q3)	25.03 (20.12–30.08)	12.31 (6.43–22.14)	23.8 (10.71–32.93)	11.8 (4.33–25.54)	0.03
SI (*μ*g/dl) 1st: Median (q1–q3)	91.84 (75.91–112.02)	86.25 (68.92–88.99)	102.13 (87.76–114.32)	86.11 (66.91–102.52)	0.38
TIBC (mg/dL) 1st: Mean ± SD	262.86 ± 46.12	242.02 ± 32.69	254.68 ± 27.38	290.54 ± 61.32	0.12
TSAT (%) 1st: Median (q1–q3)	33.68 (28.15–40.4)	33.12 (31.72–42.66)	39.94 (33.44–45.47)	30.7 (24.62–35.21)	0.22
sFt (ng/mL) 1st: Median (q1–q3)	119.3 (70.02–177.6)	100.5 (59.7–129.7)	148.6 (97.54–243.5)	87.14 (21.91–143.5)	0.14
sTfR-1 (ng/mL) 1st: Median (q1–q3)	5204.25 (4784.66–5682.16)	5852.36 (5311.59–5966.06)	5483.69 (4899.22–5776.09)	5171.77 (4957.03–5808.48)	0.29

Lab assessment at 28–30 weeks					
Serum hepcidin-25 (ng/mL) lab 2nd: Median (q1–q3)	23.9 (19.56–30.48)	12.16 (9.37–19.77)	18.94 (9.8–28.16)	12.2 (8.58–21.01)	< 0.01
SI (*μ*g/dL) 2nd: Median (q1–q3)	76.53 (58.64–90.33)	70.49 (62.38–88.88)	86.48 (52.04–122.48)	81.95 (66.19–96.54)	0.69
TIBC (mg/dL) 2nd: Median (q1–q3)	330.3 (297.66–373.18)	328.49 (318.48–384.06)	331.83 (282.35–343.58)	352.56 (337.09–403)	0.45
TSAT (%) 2nd: Median (q1–q3)	21.64 (17.2–28.84)	21.46 (19.13–27.56)	24.21 (19.73–32.71)	22.19 (18.62–27.39)	0.81
sFt (ng/mL) 2nd: Median (q1–q3)	56.5 (41.38–92.88)	36.15 (23.55–55.27)	99.69 (39.71–129.7)	76.41 (36.93–87.28)	0.25
sTfR-1 (ng/mL) 2nd: Mean ± SD	5372.04 ± 609.19	5458.33 ± 620.55	5555.59 ± 423.59	5517.76 ± 471.23	0.69

Lab assessment at 28–30 weeks before labor					
Serum hepcidin-25 (ng/mL) lab 3rd: Median (q1–q3)	27.07 (22.16–36.23)	19.69 (14.63–21.29)	10.97 (7.52–29)	15.93 (13.62–21.90)	0.02
SI (*μ*g/dL) 3rd: Median (q1–q3)	82.59 (61.77–109.23)	67.69 (45.84–120.74)	100.56 (70.66–148.97)	104.81 (87.87–133.43)	0.09
TIBC (mg/dL) 3rd: Mean ± SD	332.25 ± 71.67	316.72 ± 33.97	357.21 ± 58	343.42 ± 45.88	0.67
TSAT (%) 3rd: Median (q1–q3)	25.5 (18.62–35.64)	25.18 (14.11–35.34)	26.33 (21.01–41.53)	26.97 (26.4–43.48)	0.51
sFt (ng/mL) 3rd: Median (q1–q3)	97.49 (70.22–126.50)	57.46 (55.66–110.20)	65.45 (49.81–147.40)	135.70 (67.69–177.10)	0.55
sTfR-1 (ng/mL) 3rd: Mean ± SD	5228.95 ± 608.79	5581.73 ± 401.28	5440.33 ± 494.73	5595.02 ± 549.44	0.22

*Note:* Continuous data for normality uses ANOVA (post hoc: Bonferroni: row–column) and nonnormal use Kruskal–Wallis test (post hoc: Bonferroni correction). Categorical data uses chi-square and Fisher's exact test.

Abbreviations: GA, gestational age; Hb, hemoglobin; Hct, hematocrit; MCH, mean corpuscular hemoglobin; MCHC, mean corpuscular hemoglobin concentration; MCV, mean corpuscular volume; RBC, red blood cell; sFT, soluble ferritin; SI, serum iron; sTfR-1, soluble transferrin receptor 1; TIBC, total iron-binding capacity; TSAT, transferrin saturation.

**Table 4:** Comparisons of iron profile parameters across the three trimesters of normal pregnant women and those with thalassemia trait (excluding cases with incomplete data at any of the three time points).

**Normal**	**Trimester of pregnancy**			**p** ** value**
	**1**	**2**	**3**	
Normal pregnant women				
Serum hepcidin-25 (ng/mL) lab: Median (q1–q3)	25.03 (20.12–30.08)	23.90 (19.56–30.48)	27.07 (22.16–36.23)	0.28
SI (*μ*g/dL): Median (q1–q3)	91.84 (75.91–112.02)	76.53 (58.64–90.33)	82.59 (61.77–109.23)	< 0.01
2-1				< 0.01
TIBC (mg/dL): Mean ± SD	262.86 ± 46.12	331.43 ± 67.60	332.25 ± 71.67	< 0.01
1: Mean difference (SE)		−69.36 (8.80)	−66.78 (9.68)	< 0.01
2: Mean difference (SE)				
TSAT (%): Median (q1–q3)	33.68 (28.15–40.4)	21.64 (17.2–28.84)	25.5 (18.62–35.64)	< 0.01
2-1				< 0.01
3-1				< 0.01
sFt (ng/mL): Median (q1–q3)	119.30 (70.02–177.60)	56.50 (41.38–92.88)	97.49 (70.22–126.50)	< 0.01
2-3				< 0.01
2-1				< 0.01
sTfR-1 (ng/mL): Median (q1–q3)	5204.25 (4784.66–5682.16)	5248.77 (5003.61–5761.21)	5178.25 (4711.50–5619.45)	0.25
Pregnant women with thalassemia trait				
Serum hepcidin-25 (ng/mL) lab: Median (q1–q3)	18.22 (7.64–27.28)	15.25 (9.45–21.01)	14.63 (10.97–29)	0.54
SI (*μ*g/dL)	89.67 ± 23.79	82.71 ± 29.99	103.09 ± 34.51	0.13
TIBC (mg/dL)	262.83 ± 46.31	340.91 ± 63.43	342.97 ± 49.71	
1: Mean difference (SE)		−80.44 (14.63)	−85 (15.90)	< 0.01
2: Mean difference (SE)				
TSAT (%)	34.82 ± 9.77	24.41 ± 8.13	30.83 ± 12.27	< 0.01
1: Mean difference (SE)		12.16 (2.72)		< 0.01
2: Mean difference (SE)				
sFt (ng/mL): Median (q1–q3)	126.6 (59.7–157.5)	56.34 (35.58–99.69)	84.15 (57.46–147.4)	< 0.01
2-3				0.01
2-1				< 0.01
sTfR-1 (ng/mL): Median (q1–q3)	5498.06 (4957.03–5852.36)	5514.85 (5154.71–5829.87)	5555.29 (5102.28–5723.14)	0.41

*Note:* Nonparametric uses related-samples Friedman's two-way analysis of variance by ranks (pairwise comparisons: the Bonferroni correction for multiple tests) and for two groups that are dependent use related-samples Wilcoxon signed rank test. Parametric uses ANOVA: Bonferroni for two groups that are independent use paired samples *t*-test.

## Data Availability

The data that support the findings of this study are available from the corresponding author upon reasonable request.
